# Allelic hierarchy for *USH2A* influences auditory and visual phenotypes in South Korean patients

**DOI:** 10.1038/s41598-023-47166-w

**Published:** 2023-11-19

**Authors:** Dong Woo Nam, Yong Keun Song, Jeong Hun Kim, Eun Kyoung Lee, Kyu Hyung Park, JuHyuen Cha, Byung Yoon Choi, Jun Ho Lee, Seung Ha Oh, Dong Hyun Jo, Sang-Yeon Lee

**Affiliations:** 1 Department of Otorhinolaryngology, Chungbuk National University Hospital, Cheongju, Republic of Korea; 2 Department of Medicine, Seoul National University College of Medicine, Seoul, Republic of Korea; 3 Fight Against Angiogenesis-Related Blindness (FARB) Laboratory, Biomedical Research Institute, Seoul National University Hospital, Seoul, Republic of Korea; 4 Department of Ophthalmology, Seoul National University Hospital, Seoul National University College of Medicine, Seoul, Republic of Korea; 5 Department of Biomedical Sciences, Seoul National University College of Medicine, Seoul, Republic of Korea; 6 Department of Otorhinolaryngology-Head and Neck Surgery, Seoul National University Hospital, Seoul National University College of Medicine, 101, Daehak-ro, Jongno-gu, Seoul, 03080 Republic of Korea; 7 Department of Otorhinolaryngology-Head and Neck Surgery, Seoul National University Bundang Hospital, Seongnam, Republic of Korea; 8 Department of Anatomy and Cell Biology, Seoul National University College of Medicine, 103, Daehak-ro, Jongno-gu, Seoul, 03080 Republic of Korea; 9 Department of Genomic Medicine, Seoul National University Hospital, Seoul, Republic of Korea; 10 Sensory Organ Research Institute, Seoul National University Medical Research Center, Seoul, Republic of Korea

**Keywords:** Retinal diseases, Genetics research, Disease genetics

## Abstract

When medical genetic syndromes are influenced by allelic hierarchies, mutant alleles have distinct effects on clinical phenotypes. Genotype–phenotype correlations for Usher syndrome type 2 (USH2) suggest that the *USH2A* gene exhibits an allelic hierarchy. Here, we analyzed the phenotypes and genotypes of 16 South Korean patients with *USH2A* biallelic variants to investigate an allelic hierarchy from audiological and ophthalmological perspectives. Using whole exome and genome sequencing, 18 mutant alleles, including 4 novel alleles, were identified and implicated in *USH2A*-related disorders. Truncated alleles were linked to earlier onset of subjective hearing loss and more severe thresholds; biallelic truncated alleles had more severe effects. Truncated alleles were also associated with retinal structure degeneration and severe functional deterioration. However, younger patients (aged < 16 years) did not exhibit overt retinitis pigmentosa even when they had biallelic truncated alleles, suggesting that *USH2A*-related USH2 can mimic nonsyndromic hearing loss. For truncated alleles, there was a clear correlation between mean hearing threshold and 30-Hz flicker electroretinography implicit time. This study provides the first evidence of an *USH2A*-related allelic hierarchy among South Korean patients; our data yield valuable insights concerning the natural courses of clinical phenotypes and how genotype-based therapies may be used.

## Introduction

Usher syndrome, the leading cause of deaf-blindness, is clinically and genetically heterogeneous; it is classified into three primary subtypes based on the severity and onset of subjective hearing loss, along with the presence of vestibular dysfunction^1^. The genes responsible for Usher syndrome encode proteins of the retina and inner ear with key roles in photoreceptor maintenance, as well as sensory hair cell development and function^2,3^. These genes form the Usher interactome, which regulates clinical phenotypes via functional epistasis^4,5^. Among the disease subtypes, Usher syndrome type 2 (USH2) is the most prevalent, constituting more than half of all cases^6^. *USH2* gene loci include *USH2A* (usherin), *USH2C* (ADGRV1), and *USH2D* (whirlin)^7^. Of these, *USH2A* (OMIM 608400), located on chromosome 1q41, is implicated in up to 85% of USH2 cases^8^. Biallelic variants of *USH2A* have been linked to clinical heterogeneity in *USH2A*-related disorders, including USH2, nonsyndromic retinitis pigmentosa (RP), and nonsyndromic sensorineural hearing loss (SNHL)^9^.

Audiological manifestations of USH2 typically precede visual symptoms and signs^7^. Congenital hearing loss is detected via screening of newborn infants, which enables clinicians to identify syndromic deafness mimics (e.g., Usher syndrome) prior to the emergence of overt syndromic phenotypes^10^. The audiological profiles range in severity from moderate to severe, with a linear decrease that is particularly noticeable at high frequencies^11,12^. Additionally, USH2 causes RP with clinical symptoms that include nyctalopia and visual field constriction^13^. As the disease progresses, central vision is gradually lost, leading to blindness. Clinical signs through image evaluation include the collapse of retinal layers found in optical coherence tomography (OCT), optic nerve pallor, the development of bony spicules found in fundus photo, and the appearance of hyper-autofluorescent rings in the paramacular region^13,14^. Alterations in electroretinogram (ERG) and visual acuity are also accompanied. Because RP is generally progressive, age is a major risk factor. Although retinal degeneration can begin in childhood, disease progression may be slow and retinal phenotypes may not be noticed^15,16^; the median age at diagnosis of non-syndromic RP is ~ 35 years, when patients become aware of the problem and seek medical advice^15^. In case of syndromic RP, which *USH2A*-related consists most of the patients, the median age at diagnosis is earlier at 18–30 years^17^. However, in both types, it is still difficult for patients to notice retinal phenotypes in their childhood or adolescence.

The integration of phenotypic and genotypic information has improved the broader understanding of *USH2A*-related disorders, particularly concerning prediction of the clinical course of disease and selection of targeted therapies. The results of recent studies suggest that the *USH2A* gene exhibits an allelic hierarchy; additionally, different mutant alleles or their combinations have distinct effects on clinical phenotypes^18^. In particular, at least one nonsyndromic RP-specific allele and other missense variants with few deleterious effects are associated with isolated RP or RP with progressive hearing loss^19^, which is present in 86% of patients with *USH2A* variant-related nonsyndromic RP^9^. Moreover, at least one particular truncated variant of *USH2A* causes severe retinopathy^20^; when truncated variants of *USH2A* are combined, the resulting hearing loss may require cochlear implantation (CI)^12,21^.

Despite advances in the understanding of genotype–phenotype correlations for USH2, no studies have investigated the allelic hierarchy of *USH2A*-related disorders from audiological and ophthalmological perspectives in South Korean patients. Differences in genotypes among ethnic groups may alter the impact of mutant allele combinations, and previous data regarding allelic hierarchies may not be directly applicable to all patients with *USH2A*-related disorders^22^. Here, we investigate the links between genotypes and clinical phenotypes in South Korean patients, including audiological and ophthalmological function and retinal structure. Additionally, we evaluate the effects of truncated alleles from audiological and ophthalmological perspectives, suggesting an allelic hierarchy.

## Results

### Genotypes of *USH2A*-related disorders

We identified 14 biallelic variants in *USH2A*, either homozygous or compound heterozygous, in a trans configuration. The segregation of these variants was confirmed by Sanger sequencing. Overall, 18 mutant alleles were implicated in the diagnosis of *USH2A*-related phenotypes, including c.251G > A:p.Cys84Tyr, c.2209C > T:p.Arg737*, c.2802 T > G:p.Cys934Trp, c.4372C > T:p.Arg1578Cys, c.4858C > T:p.Gln1620*, c.7120 + 1475A > G, c.8232G > C:p.Trp2744Cys, c.85559-2A > G, c.10593del:p.Ile3532Phefs*18, c.10712C > T:p.Thr3571Met, c.10724G > T:p.Cys3575Phe, c.11156G > A:p.Arg3719His, c.12708 T > A:p.Cys4236*, c.13112_13115del:p.Gln4371Argfs*19, c.14134-3169A > G, c.13964 T > C:p.Leu4655Pro, c.14835del:p.Val4946Trpfs*4, and c.14911C > T:p.Arg4971* (Table 1 and Supplementary Fig. S1). The amino acid residues were highly conserved in orthologs from several species (Supplementary Fig. S1), a finding consistent with the corresponding high Genomic Evolutionary Rate Profiling (GERP + +) score. Two variants, c.2802 T > G:p.Cys934Trp and c.8559-2A > G variants, were present more than twice. Consistent with our findings, these two variants have been recurrently identified and are recognized as East Asian-specific founder and mutational hotspot alleles associated with *USH2A* gene^23^. Four variants were novel, including two missense variants, one frameshift variant, and one deep intronic variant. The frameshift variant p.Ile3532Phefs*18 had a premature termination codon, which resulted in nonsense-mediated mRNA decay. This variant was extremely rare in population databases, including the Korean Reference Genome Database, which is based on genetic data from South Koreans. Furthermore, co-segregation analyses confirmed the presence of this variant in trans with a known pathogenic variant (c.8559-2A > G). Based on the American College of Medical Genetics and the Association for Molecular Pathology (ACMG/AMP) guidelines^24,25^, this novel frameshift variant was classified as pathogenic (PVS1, PM2, PM3). The novel missense variants p.Cys3575Phe and p.Leu4655Pro, located in the fibronectin type III-20 and fibronectin type III-32 domains, respectively, were also extremely rare in population databases and occurred in trans with a pathogenic variant. In silico analysis, using Combined Annotation Dependent Depletion^26^, predicted that these variants would be pathogenic. Based on the ACMG/AMP guidelines^24,25^, the novel missense variants p.Cys3575Phe and p.Leu4655Pro were classified as likely pathogenic (PM2, PM3, PM5, PP4) and variants of uncertain significance (PM2, PM3, PP4), respectively. Furthermore, two deep intronic variants, c.14134-3169A > G and c.7120 + 1475A > G, were identified by trio-based whole-genome sequencing (WGS) and bioinformatics analyses. The previously reported c.14134-3169A > G variant in intron 64 led to pseudo-exon activation, which resulted in a truncated protein^27^. The novel deep intronic variant c.7120 + 1475A > G in intron 37 was predicted to introduce pseudo-exon activation. This newly inserted pseudo-exon had a premature termination codon, which probably resulted in a truncated translation product. We have reclassified the conflicting or variants of uncertain significance documented in the ClinVar database, using the ACMG/AMP guidelines^24,25^. Upon evaluation using either the ClinVar database or the ACMG/AMP guidelines, 16 of the 18 variants (with the exceptions of c.251G > A:p.Cys84Tyr and c.13964 T > C:p.Leu4655Pro) were determined as either pathogenic or likely pathogenic (Table 1). The genotype information of the disease-causing *USH2A* variants, mapped to both hg19 and hg38, is described in Supplementary Table 1.

**Table 1:** *USH2A* variants in the current study and its pathogenicity prediction analysis.

Genomic position (GRCh37/hg19)	HGVS		Location (Exon/domain)	In-silico Prediction		Allele Frequency		Clinvar database		ACMG/AMP 2018 guideline	
	Nucleotide change	Amino Acid change		CADD	REVEL	Splice AI Maxent	KOVA KRGDB	gnomAD	Classificatioin	Criteria	Classification
Chr1:216595428C-T	c.251G > A	p.Cys84Tyr	Exon 2/Absent	22.90	0.483	NA	ND	ND	Uncertain significance	PM2, PM3, PP4	Uncertain significance
Chr1:216420527G-A	c.2209C > T	p.Arg737*	Exon 13/Laminin EGF like 4	36.00	NA	NA	ND	exome (8.151e−06) genome(ND)	Pathogenic (PMID10729113)		
Chr1:216419934A-C	c.2802 T > G	p.Cys934Trp	Exon 13/Laminin EGF like 8	25.20	0.849	NA	0.00291206	exome(0.0002111)gnome(0.0001274)	Conflicting Pathogenic(10); Likely pathogenic(3); Uncertain significance(3)	PS1, PM3, PP3, PP4	Likely pathogenic
Chr1:216270451G-A	c.4732C > T	p.Arg1578Cys	Exon 22/Laminin G-like 1	24.20	0.695	NA	ND	exome(1.593e−05) genomes(3.185e−05)	Pathogenic (PMID22135276)		
Chr1:216262383G-A	c.4858C > T	p.Gln1620*	Exon 23/Laminin G like 1	37.00	NA	NA	ND	ND	Likely pathogenic (PMID21697857)		
Chr1:215963842 T-C	c.7120 + 1475A > G	p.?	Intron 37	22.40	NA	DG(0.25) DL(0.00) MAXENT (NA)	ND	ND	ND	PVS1, PM2, PM3	Pathogenic
Chr1:216052432C-G	c.8232G > C	p.Trp2744Cys	Exon 42/Fibronectin type III 14	29.30	0.651	NA	ND	exome(8.296e−06) genome(ND)	Pathogenic (PMID21686329)		
Chr1:216051224 T-C	c.8559-2A > G	p.?	Intron 42	34.00	NA	AG(0.00) AL(0.99) /MAXENT (2.13)	ND	exome(3.186e−05) genome(ND)	Pathogenic (PMID10729113)		
Chr1:215955530A-T	c.10593del	P.Ile3532Phefs*18	Exon 54/Fibronectin type III 20	ND	NA	NA	ND	ND	ND	PVS1, PM2, PM3	Pathogenic
Chr1:215955412G-A	c.10712C > T	p.Thr3571Met	Exon 54/Fibronectin type III 20	25.30	0.622	NA	ND	exome(1.194e−05) gemone(ND)	Pathogenic (PMID19683999)		
Chr1:215955400C-A	c.10724G > T	p.Cys3575Phe	Exon 54/Fibronectin type III 20	28.00	0.367	NA	ND	ND	ND	PM2, PM3, PM5, PP4	Likely pathogenic
Chr1:215933077C-T	c.11156G > A	p.Arg3719His	Exon 57/Fibronectin type III 22	25.80	0.334	NA	0.00029121	exome(5.283E−05) genome(9.689E−05)	Pathogenic (PMID25133613)		
Chr1:215848545A-T	c.12708 T > A	p.Cys4236*	Exon 63/Fibronectin type III 27	41.00	NA	NA	ND	ND	Pathogenic (PMID21593743)		
Chr1:215848137CATT-C	c.13112_13115del	p.Gln4371Argfs*19	Exon 63/Fibronectin type III 29	33.00	NA	NA	ND	exome(1.221e−05) genome(3.184e−05)	Pathogenic/Likely pathogenic (PMID28894305)		
Chr1:215844483A-G	c.13964 T > C	p.Leu4655Pro	Exon 64/Fibronectin type III 32	27.80	0.529	NA	ND	ND	ND	PM2, PM3, PP4	Uncertain Significance
Chr1:215827321 T-C	c.14134-3169A > G	p.?	Intron 64	3.30	NA	AG(0.02) AL(0.00) MAXTENT (NA)	ND	ND	Pathogenic (PMID29196752)		
Chr1:215814032CA-A	c.14835del	p.Val4946Trpfs*4	Exon 68/Absent	ND	NA	NA	ND	ND	Pathogenic (PMID21697857)		
Chr1:215813957G-A	c.14911C > T	p.Arg4971*	Exon 68/Absent	48.00	NA	NA	ND	exome(ND) genome(ND)	Pathogenic (PMID10729113)		

Abbreviations: ND, not determined; NA, not available.

Refseq transcript accession number NM_206933.4; Refseq protein accession number NP_996816.3

HGVS: Human Genome Variation Society (https://www.hgvs.org/).

CADD: Combined Annotation Dependent Depletion (https://cadd.gs.washington.edu/ ).

REVEL: Rare Exome Variant Ensemble Learner (https://sites.google.com/site/revelgenomics/).

SpliceAI (https://spliceailookup.broadinstitute.org/).

KRGDB: Korean Reference Genome Database (http://coda.nih.go.kr/coda/KRGDB/index.jsp).

KOVA: Korean Variant Archive (https://www.kobic.re.kr/kova/).

gnomAD: The Genome Aggregation Database (https://gnomad.broadinstitute.org/).

ACMG/AMP 2018 guideline (http://wintervar.wglab.org/).

*Analysis of ACMG/AMP guidelines for novel variants as well as conflicting or variants of uncertain significance documented in the Clinvar database.

To investigate the allelic hierarchy of *USH2A*-related disorders, we categorized patients with *USH2A* biallelic variants into three groups based on their truncated and nontruncated mutant alleles: group 1 consisted of individuals with two truncated mutant alleles; group 2 consisted of individuals with one truncated and one nontruncated mutant allele; and group 3 consisted of individuals with two nontruncated mutant alleles (Supplementary Table 2).

### Audiological phenotypes and allelic hierarchy

**Figure 1:**
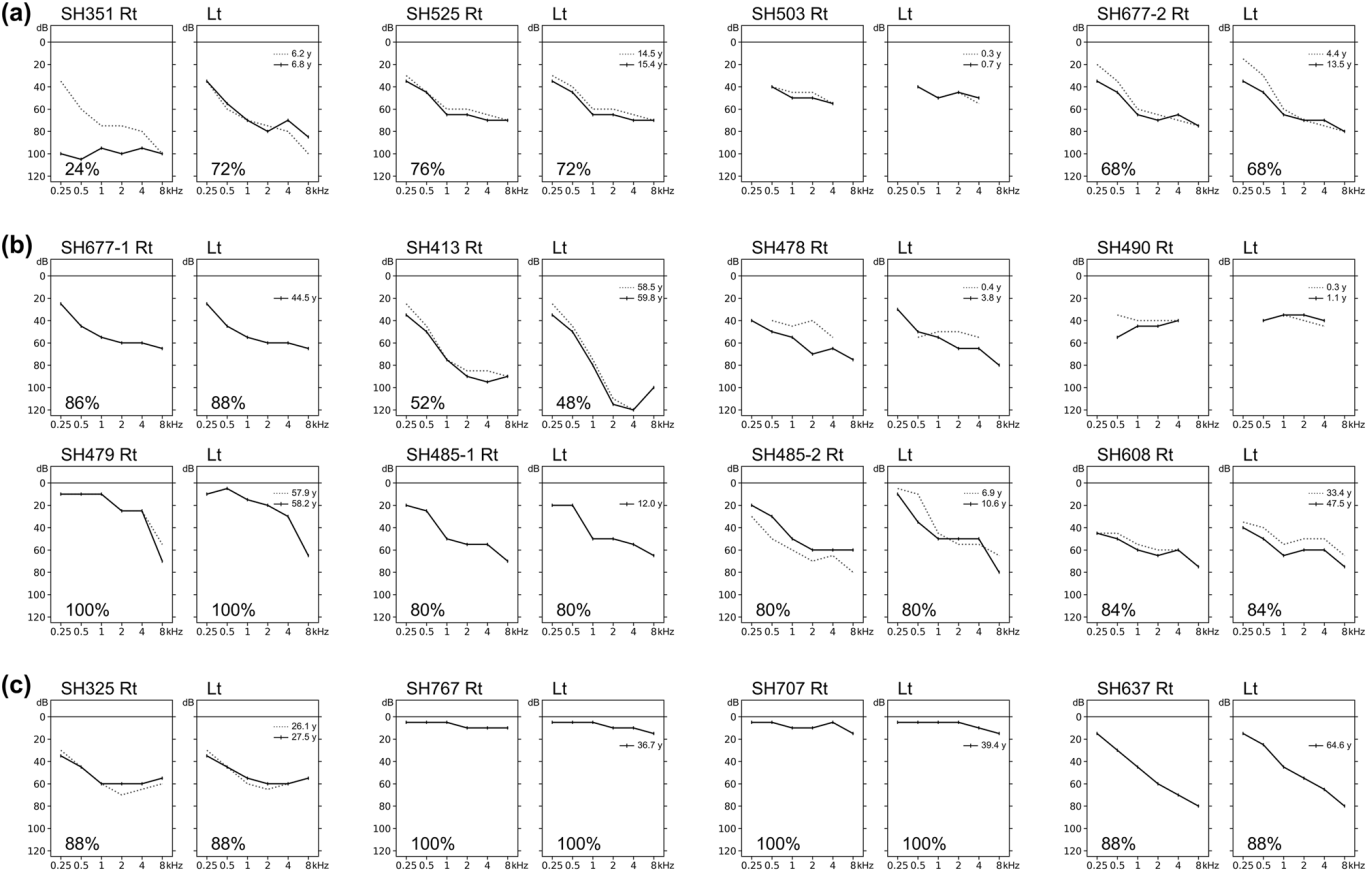
Audiograms of 16 patients with biallelic Usher syndrome type 2A (*USH2A*) variants. (**a**) Four patients in group 1. (**b**) Eight patients in group 2. (**c**) Four patients in group 3. Speech discrimination scores are indicated in lower left corners, except when the test was not performed because the patient was too young.

The audiological characteristics of our cohort are shown in Table 2; the corresponding audiograms are shown in Fig. 1. Based on the latest audiograms, three patients (18.8%) had normal hearing (SH479, SH767, and SH707), indicating nonsyndromic RP. The remaining 13 patients (81.2%) had varying degrees of SNHL; this was typically mild-to-moderate at low frequencies and severe-to-profound at high frequencies. Among these 13 patients, six had moderate SNHL (46.2%), five had moderately severe SNHL (38.5%), and two had severe SNHL (15.4%). Subjective onset of hearing loss was congenital in six (46.2%), prelingual in two (15.4%), and postlingual in five (38.5%) patients. All but one patient (SH351) had symmetric bilateral hearing loss that deteriorated at higher frequencies (10 of 12, 83.3%), or they exhibited a horizontal trace pattern (2 of 12, 16.7%). Consistent with the findings in previous studies^21^, during the follow-up period, which ranged from 0.3 to 14.1 years with a median duration of 1.3 years, all 11 patients who underwent hearing evaluations had milder progressive hearing loss. One patient (SH351) with biallelic truncated variants underwent unilateral CI in their worse ear before the age of 13 years. Neither congenital cytomegalovirus infection nor inner ear anomalies underlying asymmetric hearing loss were identified in this patient (SH351). Most patients with confirmed hearing loss used hearing aids.

**Table 2:** Demographics and clinical phenotypes in our *USH2A* cohort.

Subject	Sex/age	Genotype	Zygosity	Group	Auditory Phenotype					Visual Phenotype			
					Onset	Hearing threshold (dB HL; Rt/Lt)	Speech Discrimination (%; Rt/Lt)	Configuration	Symmetry	Visual acuity (logMAR; OD/OS)	Cone ERG response	Visual field (Maximum isopter)	OCT (Junctional complex)
SH351	F/6	c.14911C > T:p.Arg4971*	Comp het	1	Congenital^a^	98/68	24/72	Flat (Rt), sloping (Lt)	No		Excluded		
		c.2209C > T:p.Arg737*											
SH525	F/14	c.12708 T > A:p.Cys4236*	Homo	1	Prelingual^b^	61/61	76/72	Sloping	Yes		Excluded		
SH503	M/4 M	c.14134-3169A > G	Comp het	1	Congenital	48/46	NA	Flat	Yes		Excluded		
		c.14835del:p.Val4946Trpfs*4											
SH677-1	F/12	c.8559-2A > G:p.?	Comp het	1	Prelingual	61/62	68/68	Sloping	Yes		Excluded		
		c.10593del:p.Ile3532Phefs*18											
SH413	F/57	c.251G > A:p.Cys84Tyr	Comp het	2	Postlingual^c^ (since 30’s )	77/91	52/48	Sloping	Yes	1.398/1.398	No	10	Absent
		c.13112_13115del:p.Gln4371Argfs*19											
SH478	M/5	c.8559-2A > G:p.?	Comp het	2	Congenital	60/58	NA	Sloping	Yes		Excluded		
		c.11156G > A;p.Arg2719His											
SH490	M/5 M	c.2802 T > G:p.Cys934Trp	Comp het	2	Congenital	46/37	NA	Flat	Yes		Excluded		
		c.4858C > T:p.Gln1620*											
SH479	F/57	c.14835del:p.Val4946Trpfs*4	Comp het	2	NA^d^	17/17	100/100	Sloping	Yes	0.155/0	Yes	12	Present
		c.13964 T > C:p.Leu4655Pro											
SH485-1	M/7	c.10712C > T:p.Thr3571Met	Comp het	2	Congenital	50/46	80/80	Sloping	Yes		Excluded		
		c.7120 + 1475A > G											
SH485-2	F/5	c.10712C > T:p.Thr3571Met	Comp het	2	Congenital	46/43	80/80	Sloping	Yes		Excluded		
		c.7120 + 1475A > G											
SH608	M/47	c.2802 T > G:p.Cys934Trp	Comp het	2	Postlingual (since 10’s )	58/58	84/84	Sloping	Yes	1/1.699	No	13	Absent
		c.13112_13115del:p.Gln4371Argfs*19											
SH677-2	M/33	c.8559-2A > G:p.?	Comp het	2	Postlingual (since 10’s )	55/55	86/88	Sloping	Yes	0.398/0.398	No	13	Absent
		c.251G > A:p.Cys84Tyr											
SH325	M/27	c.11156G > A:p.Arg3719Hisx	Comp het	3	Postlingual (since 20’s )	56/55	88/88	Sloping	Yes	0.222/0.523	No	10	Present
		c.251G > A:p.Cys84Tyr											
SH767	M/34	c.2802 T > G:p.Cys934Trp	Homo	3	NA	7/7	100/100	Flat	Yes	2/2	No	4	Absent
SH707	M/39	c.4372C > T:p.Arg1578Cys	Comp het	3	NA	7/6	100/100	Flat	Yes	0.046/0.046	Yes	80	Present
		c.2802 T > G:p.Cys934Trp											
SH637	F/64	c.11156G > A:p.Arg3719His	Comp het	3	Postlingual (since 30’s )	51/47	88/88	Sloping	Yes	0/0.046	Yes	53	Present
		c.10724G > T:p.Cys3575Phe											

a: congenital onset, cases identified as refer in newborn hearing screening.

b: prelingual onset, cases in which hearing aids were worn before school age, that is, before the age of 7.

c: postlinigual onset, cases with subjective hearing loss onset.

d: NA, cases not documented for hearing loss on the audiogram.

Rt: right; Lt: left; comp het: compound heterozygote; homo: homozygote.

**Figure 2:**
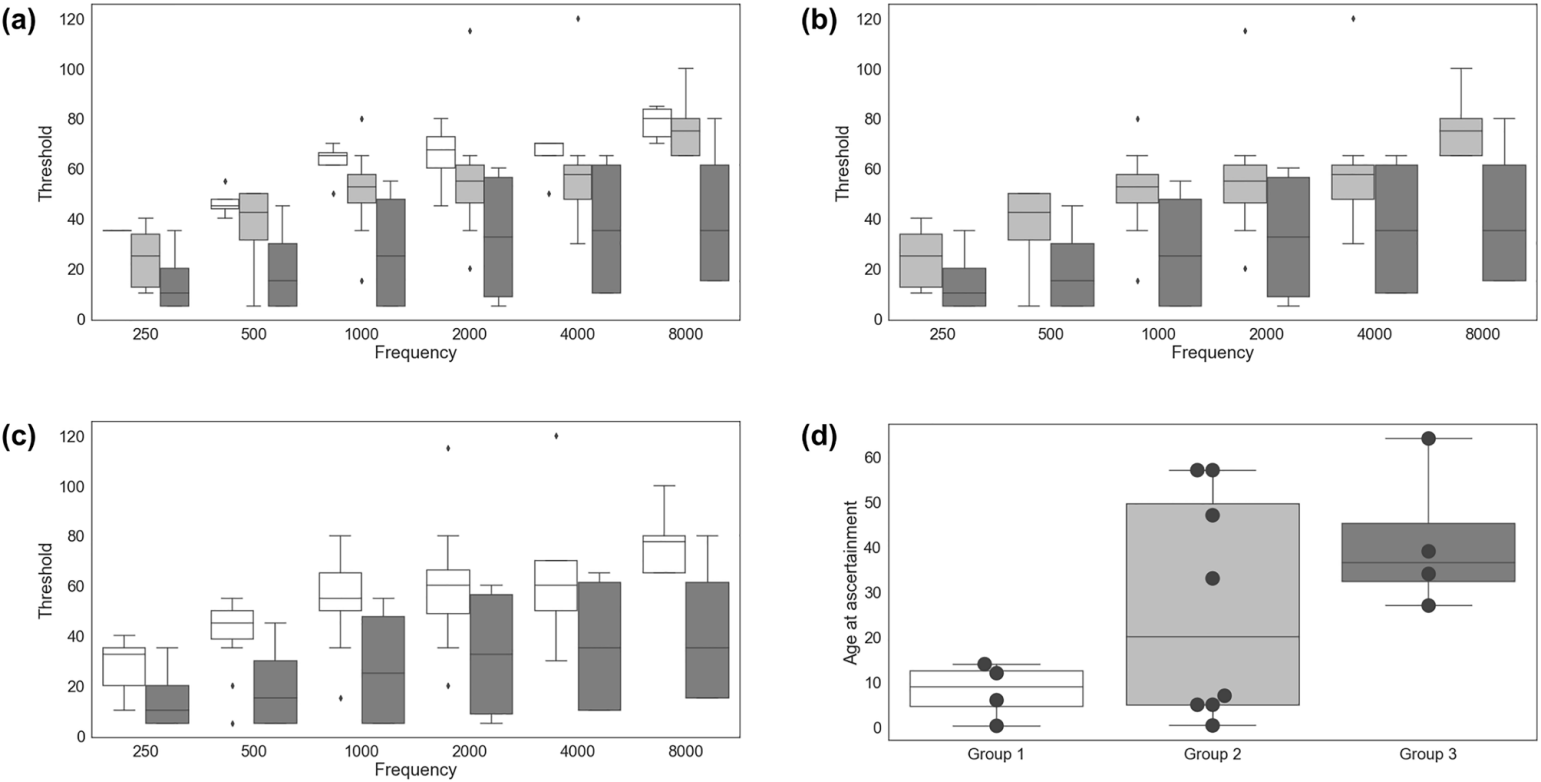
Hearing thresholds and ages at ascertainment, stratified by group. (**a**) Hearing thresholds, stratified by group (1 vs. 2 vs. 3). (**b**) Hearing thresholds, stratified by group (1 + 2 vs. 3). (**c**) Hearing thresholds, stratified by group (2 vs. 3). (**d**) Age at ascertainment, stratified by group (1 vs. 2 vs. 3).

The mean hearing thresholds were 59.7 dB, 51.1 dB, and 29.1 dB in group 1 (two truncated alleles), group 2 (one truncated and one nontruncated allele), and group 3 (two nontruncated alleles), respectively (Fig. 2a). The thresholds were consistently highest across frequencies in group 1 and lowest in group 3. We recorded mean hearing thresholds for group 1 versus group 2 versus group 3 at 250 Hz (35.0 vs. 24.3 vs. 15.0, *p* = 0.015 by Kruskal–Wallis rank sum test), 500 Hz (46.3 vs. 36.9 vs. 20.0, *p* = 0.021 by Kruskal–Wallis rank sum test), 1000 Hz (62.5 vs. 50.6 vs. 27.5, *p* = 0.005 by Kruskal–Wallis rank sum test), 2000 Hz (65.0 vs. 56.9 vs. 32.5, *p* = 0.024 by Kruskal–Wallis rank sum test), 4000 Hz (65.0 vs. 60.0 vs. 36.3, *p* = 0.032 by Kruskal–Wallis rank sum test), and 8000 Hz (78.3 vs. 75.7 vs. 41.3, *p* = 0.013 by Kruskal–Wallis rank sum test). Patients with at least one truncated allele (groups 1 and 2) tended to have more severe hearing loss than patients with biallelic nontruncated alleles (group 3; Fig. 2b); patients with one truncated allele had higher hearing thresholds than patients with biallelic nontruncated alleles (Fig. 2c). Nonsyndromic RP was more common among patients in group 3 than among patients in the other groups. All patients in group 1 had congenital or prelingual onset of subjective hearing loss, whereas most patients in the other groups (except three patients in group 2) had postlingual onset or normal hearing. Moreover, patients in group 1 were younger at ascertainment than patients in groups 2 and 3, although this difference was not statistically significant (Fig. 2d, p = 0.132 by Kruskal–Wallis rank sum test). In summary, our audiological data suggest that truncated alleles are associated with more severe phenotypes for both the extent and severity of progressive hearing loss; these effects were greater for biallelic truncated alleles, implying that the *USH2A* gene exhibits an allelic hierarchy from an audiological perspective.

### Retinal phenotypes and allelic hierarchy

As previously reported, age has a strong effect on retinal phenotypes. Patients under 16 years of age did not report ophthalmological symptoms prior to clinical evaluation. Previous report shows that the onset of syndromic RP, although earlier than non-syndromic RP, is still delayed after adolescence^15–17^. Eight of the patients were under 16 years old, and their phenotypes mimicked nonsyndromic hearing loss. Therefore, eight patients aged < 16 years were excluded from analyses of retinal phenotypes; this led to exclusion of all participants from group 1. The remaining patients in group 2 were 33, 47, 57, and 57 years old; the remaining patients in group 3 were 30, 37, 40, and 65 years old. Visual acuity, electroretinography (ERG), Goldmann visual field (GVF), optical coherence tomography (OCT), fundus photographs, and fundus autofluorescence (FAF) assessments were used to evaluate each patient’s retinal phenotype.

**Figure 3:**
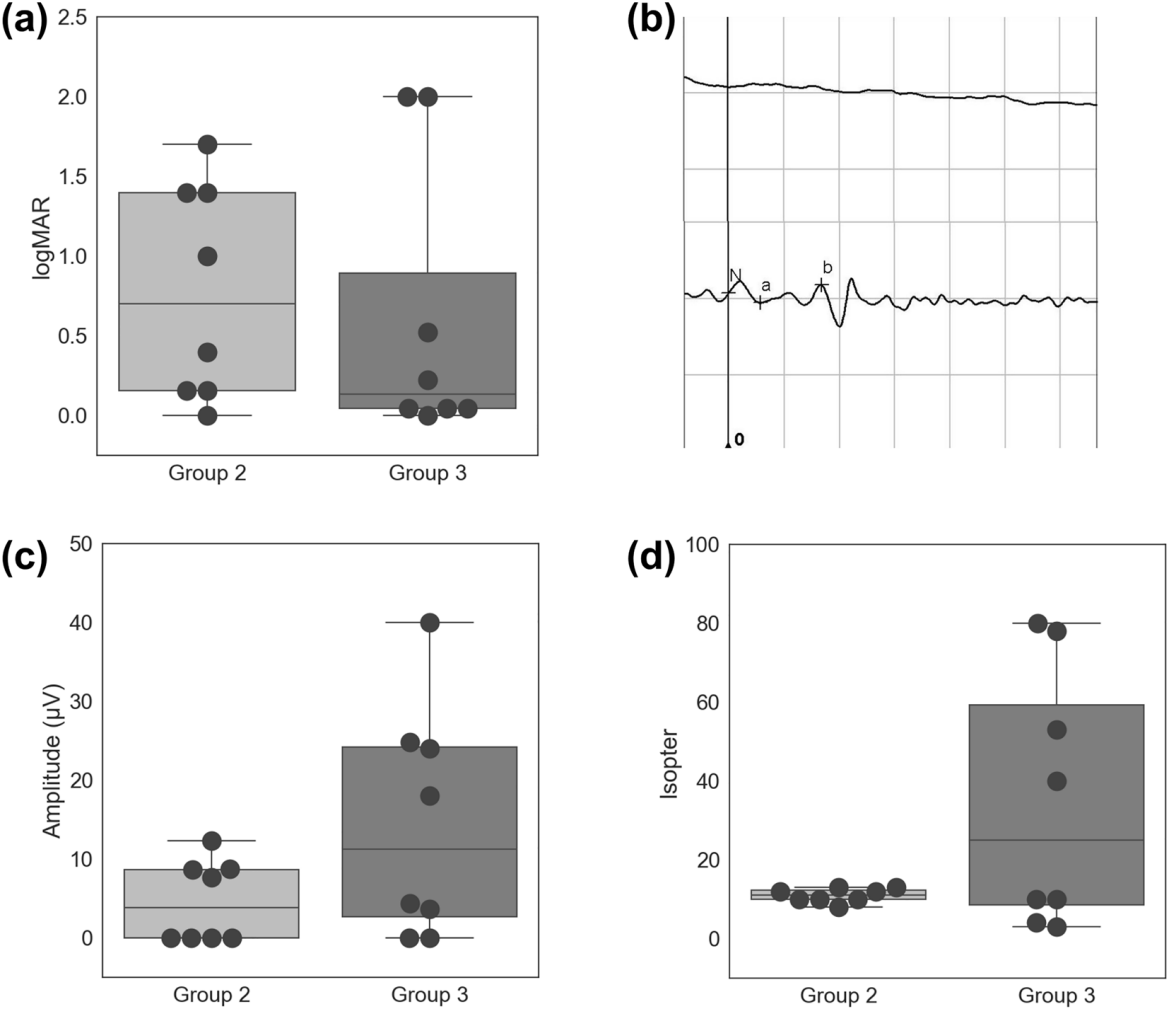
Comparison of functional consequences of retinal degeneration between patients of Groups 2 and 3. (**a**) Visual acuities in logMAR scale in Group 2 and Group 3 patients. (**b**) Representative scotopic 3.0 electroretinograms of Group 2 (above) and Group 3 (below) patients. Each column of the x-axis represents 25 ms, and each column of the y-axis represents 100 mV. (**c**) Response amplitudes in ERG 30 Hz flicker tests in Group 2 and Group 3 patients. (**d**) Maximal isopters in Goldmann perimetry in Group 2 and Group 3 patients. logMAR, logarithm of the minimum angle of resolution; ERG, electroretinography.

The median best-corrected visual acuity values, determined using the logarithm of the minimum angle of resolution (logMAR) scale, showed that there was no significant difference in retinal function among the groups (group 2: median = 0.699, range = 0–1.699; group 3: median = 0.134, range = 0–2; Fig. 3a; *p* = 0.597 by Wilcoxon rank sum test). All patients included in this study did not have other ophthalmological diseases, which may affect visual acuity, such as cataract or preretinal fibrosis, at the time of evaluation. Additionally, we evaluated ERG responses for each patient. The ERG response of many patients with RP is undetectable, which indicates reduced or no function^28^. We measured residual responses using three different ERG settings: 0.01 scotopic ERG for a rod response, 3.0 scotopic ERG for a combined rod and cone response, and a photopic test for a cone response. The response of 0.01 scotopic ERG in all patients were undetectable. These results are consistent with the findings in previous reports, in which the progression of RP initially affected rod function^13,14^. Only one-fourth of the patients in group 2 exhibited residual cone function, whereas half of the patients in group 3 exhibited a cone response. Representative graphs for 3.0 scotopic ERG are shown in Fig. 3b. The 30-Hz flicker test evaluates cone function and was used to investigate RP in a previous study^29^. Group 3 patients exhibited higher response amplitudes compared with group 2 patients, indicating that cone function had been more effectively preserved in group 3 patients, but it was statistically insignificant (*p* = 0.215 by Wilcoxon rank sum test; Fig. 3c). As ERG is often altered in RP patients before other signs or symptoms appear, the ERG of excluded patients under age 16 were also analyzed. With the exception of one patient in group 2, 0.01 scotopic ERG response was undetectable. The photopic ERG response was found in all excluded patients due to age, indicating the preservation of cone function in younger ages. The visual fields of all patients in group 2 and two patients in group 3 were restricted to a central area. However, the other two patients in group 3 had relatively preserved visual fields (Fig. 3d). These ERG and GVF results reveal that patients with *USH2A*-related RP who have truncated alleles tend to exhibit more severe functional retinal degeneration.

**Figure 4:**
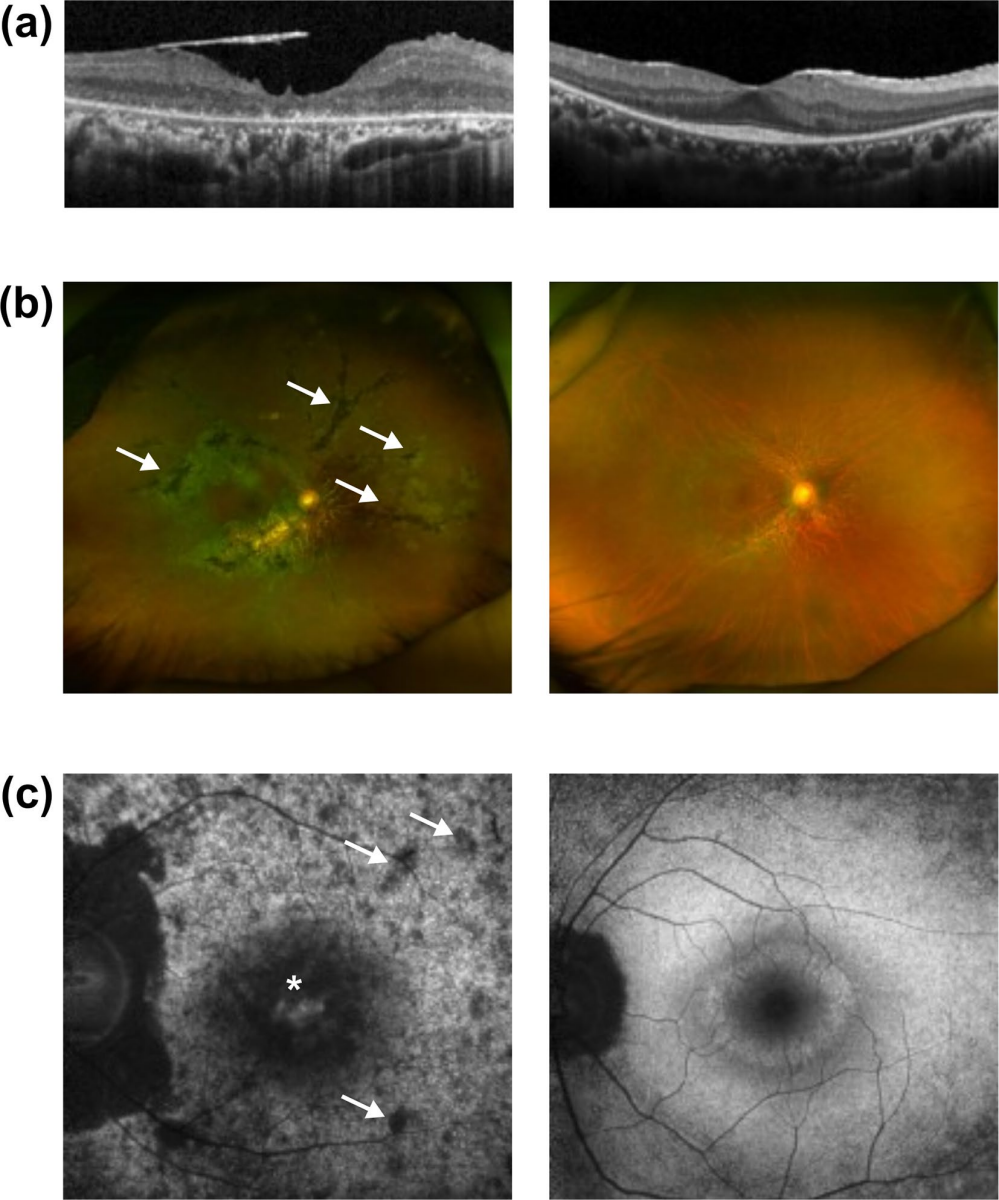
Comparison of structural consequences between patients of Groups 2 and 3. (**a**) Representative images of optical coherence tomography of Group 2 (left) and Group 3 (right) patients. (**b**) Representative wide fundus photographs of Group 2 (left) and Group 3 (right) patients. The arrows indicate bony spicules, an indication of retinal degeneration. (**c**) Representative image of fundus autofluorescence of Group 2 (left) and Group 3 (right) patients. The asterisk (*) indicates the hyperfluorescent fovea and arrows indicate hypo-fluorescent mottles in the paramacular region.

For evaluation of retinal structure, OCT was used to examine the integrity of the junctional complex, which consists of an interdigitation zone, an ellipsoid zone, and an external limiting membrane^30^. Only one-fourth of the patients in group 2 exhibited conserved junctional complexes, compared with three-fourths of the patients in group 3 (Fig. 4a). Additionally, we compared retinas from patients in groups 2 and 3 with respect to bony spicules and waxy optic discs, which indicate retinal degeneration^31^. Fundus photographs revealed that group 2 patients had more bony spicules, and these were distributed more widely. Although this difference may occur from the difference of duration of the disease, the number and distribution, combined with overall retinal intactness, supports that group 2 patients show more severe phenotypes in a qualitative manner. In addition, the spicules in group 2 patients were thicker and wider in size and more apparent overall. Furthermore, all four patients in group 2 exhibited waxy optic discs, indicating progressing pallor; only one-fourth of the patients in group 3 exhibited similarly degenerate optic discs (Fig. 4b). Patients in group 2 also exhibited more paramacular mottling in FAF images than patients in group 3. Patients from both groups also underwent evaluation of hyper-autofluorescent rings (i.e., Robson–Holder rings), which are characteristic of RP^32,33^. The ring structures present in group 3 patients were less obvious than the structures present in group 2 patients or were completely absent. In addition, although being fainter, the integrity of the ring structure was more intact in group 2 patients, indicating the absence of the hyper-autofluorescent ring is not due to further advance of degeneration. Indicating less severe phenotypes in group 3 (Fig. 4c). Overall, these OCT, fundus, and FAF results indicate that patients with truncated alleles tend to exhibit more severe structural disruption in their retinas.

### Correlations between audiological and ophthalmological phenotypes

**Figure 5:**
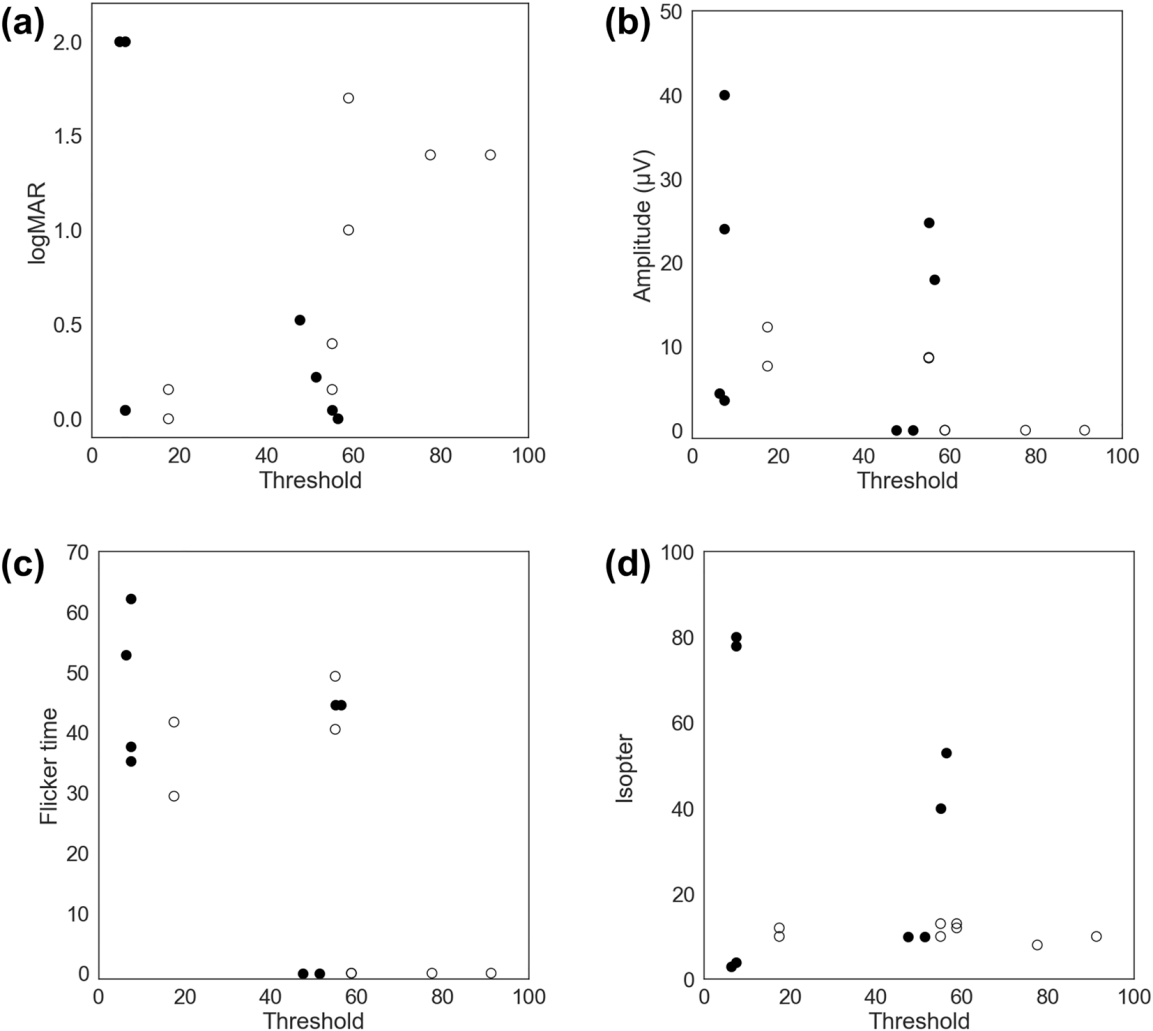
Relationships between hearing threshold and ophthalmological phenotypes. (**a**) Hearing threshold and logMAR visual acuity. (**b**) Hearing threshold and 30-Hz flicker ERG amplitude. (**c**) Hearing threshold and 30-Hz flicker ERG implicit time. (**d**) Hearing threshold and the maximum isopter produced by Goldmann visual field evaluation. Groups 2 and 3 values are represented by white (○) and black circles (●), respectively.

In this study, we analyzed the correlation between audiological and ophthalmological phenotypes in eight suitable patients (groups 2 and 3; Fig. 5). Although mean hearing threshold was not correlated with logMAR visual acuity (Spearman correlation coefficient = 0.137, *p* = 0.613), 30 Hz flicker ERG amplitude (Spearman correlation coefficient =  − 0.456, *p* = 0.076), or the maximum isopter on GVF evaluation (Spearman correlation coefficient = 0.050, *p* = 0.853), there was a notable correlation between mean hearing threshold and 30-Hz flicker ERG implicit time (Spearman correlation coefficient =  − 0.525, *p* = 0.037). In particular, the correlation had distinct effects on the presence of a truncated allele. Group 2 patients exhibited a negative correlation (Spearman correlation coefficient =  − 0.775, *p* = 0.024), whereas group 3 patients exhibited no discernible correlation (Spearman correlation coefficient =  − 0.271, *p* = 0.515). In the same vein, subgroup analysis of Group 2 revealed correlations between hearing threshold and visual acuity (Spearman correlation coefficient = 0.859, *p* = 0.006) and between hearing threshold and 30 Hz flicker ERG amplitude (Spearman correlation coefficient =  − 0.827, *p* = 0.011).

## Discussion

This study provides a comprehensive analysis of the clinical features of *USH2A*-related disorders and presents evidence for an allelic hierarchy in South Korean patients. Our results show that patients with at least one truncated variant exhibit more severe audiological and retinal phenotypes, compared with patients who have biallelic nontruncated variants. Notably, this trend is even greater in patients with biallelic truncated variants, suggesting that the severity of disorders is determined by the presence and number of truncated mutant alleles. Additionally, there was a clear correlation between mean hearing threshold and 30-Hz flicker ERG implicit time, depending on the presence of a truncated allele. These findings have important clinical implications because data regarding genotype–phenotype correlations and allelic hierarchies can be used to predict the natural course of disease and to guide the use of gene therapies.

Several medical genetic syndromes involve allelic hierarchies, including cystic fibrosis, hemophilia, neurofibromatosis type I, and Usher syndrome^12,34–36^. Phenotype severity depends on the particular mutant allele and its effect on protein function. The *USH2A* gene exhibits an allelic hierarchy; there are tissue-specific genotype–phenotype correlations for various clinical manifestations of *USH2A*-related disorders, including hearing loss, RP, and olfactory function^37–39^. The results of previous studies suggested an allelic hierarchy for nonsyndromic RP that was associated with the *USH2A* gene, particularly for the allelic variant p.Cys759Phe. Those studies showed that patients who were heterozygous for p.Cys759Phe and had an additional truncated or USH2-related missense variant tended to exhibit earlier onset and more severe clinical phenotypes, compared with patients who were homozygous for p.Cys759Phe^19^. Furthermore, truncated alleles of *USH2A* are more likely to have deleterious effects on protein structure and function; they may result in more severe clinical phenotypes, compared with missense or other nontruncated variants. These phenomena occur because truncated variants often lead to premature protein termination, resulting in a loss of function or a mutant protein that cannot fulfill its normal physiological role^40^. Recent allelic hierarchy data suggest that the presence of biallelic truncated alleles is likely to cause severe audiological phenotypes, such as hearing loss. For example, homozygous truncated c.2299delG allelic variants are associated with severe and progressive hearing loss in patients with *USH2A*-related disorders^21^. Findings in our previous study suggested that a particular combination of biallelic truncated alleles of *USH2A* could result in more severe audiological phenotypes and a greater need for CI, compared with other allele combinations^12^. The present study provides further support for this observation because we identified a patient (SH351) with two truncated alleles who exhibited severe-to-profound progressive hearing loss and who underwent CI before the age of 13 years. Therefore, an understanding of the potential impacts of allelic hierarchies on audiological phenotypes is important for personalized audiological rehabilitation. The results of this study improve the broader understanding of allelic hierarchies from an audiological perspective; they reveal that the presence of at least one truncated allele can result in earlier onset of subjective hearing loss and more severe thresholds. We also noted greater effects for biallelic truncated alleles, an observation that is consistent with the dose-dependent effect on hearing loss severity identified in the RUSH2A study^37^. Furthermore, the allelic hierarchy was not restricted to one particular truncated allele, indicating that different truncated variants of *USH2A* may have similar effects on audiological phenotypes. However, the present study did not evaluate missense alleles in the N-terminal laminin domain of *USH2A*; two of these alleles are associated with USH2 but not with nonsyndromic RP. Therefore, compared with truncated alleles, the presence of these missense alleles may have different effects on clinical presentation^41^.

For the 10 patients identified with non-congenital hearing loss, we made an analysis to determine the rate of auditory decline since birth (i.e., hearing loss progression, dB/year). We then assessed the correlation coefficient between the hearing loss progression and subjective onset of hearing loss (prelingual, postlingual, and normal). This analysis yielded a Spearman’s rank correlation coefficient of − 0.669 (*p* = 0.035), suggesting a inverse relationship. In other words, this finding poses that an accelerated rate of hearing loss progression is associated with an earlier onset of subjective hearing loss.

Our ophthalmological findings also suggest that the *USH2A* gene exhibits an allelic hierarchy. All ophthalmological examinations revealed differences in the severity of RP symptoms between patients with and without truncated alleles. Functional measurements and structural studies yielded similar results, showing that patients with truncated alleles exhibited accelerated structural degeneration leading to loss of visual function, as previously reported^42^. The confounding effects of age on RP severity have been reported for patients with various genetic backgrounds, regardless of specific mutations^15,16^. However, an older patient from group 3 (SH637) exhibited superior functional and structural preservation compared with a younger patient from group 2 (SH608). This example suggests that the genetic influence of *USH2A* is sufficiently strong to control the natural progression of RP. One patient from group 3 (SH707) exhibited a particularly severe retinal phenotype, with collapsed retinal structure and significant losses affecting both visual field and visual acuity. This patient had the previously unreported c.4372C > T variant, which clearly has severe effects on retinal structure and function. Consequently, there are exceptions to the allelic hierarchy, such that some nontruncated variants elicit severe phenotypes. Nontruncated variants reportedly have various levels of pathogenicity in *USH2A*-related Usher syndrome. A more elaborate hierarchical system based on both truncation number and nontruncated variant pathogenicity may provide a better understanding of the relationship between genotypes and phenotypes.

Importantly, RP is strongly associated with age^15,16^. Even patients with pathogenic gene variants may not notice the onset of RP for an extended interval. Therefore, the lack of correlation between auditory and visual phenotypes may be related to differences in the onset and progression of these phenotypes. Despite a few exceptions, the allelic hierarchy between groups was clearly based on the presence of truncating alleles. Future studies, including long-term follow-up of younger patients with Usher syndrome, may provide further insights regarding auditory–visual correlations and the confounding effects of age.

An understanding of allelic hierarchies may enable clinicians to predict the course of clinical phenotypes associated with *USH2A*-related disorders. This information may also be used to develop personalized monitoring plans and treatment strategies tailored to the truncated or nontruncated mutant alleles involved. Therefore, early identification of *USH2A* biallelic variants is particularly important for patients with these disorders; this information should facilitate timely interventions and may improve outcomes^10^. Newborn infant screening approaches are crucial for the identification of SNHL and the underlying genetic causes of hearing loss. Screening facilitates early rehabilitation of affected individuals. Our studies show that retinal phenotypes may not be present when hearing loss is first observed in pediatric patients with USH2, even if the patient has two truncated *USH2A* alleles. Therefore, *USH2A*-related USH2 can mimic nonsyndromic hearing loss. Nonetheless, the allelic hierarchy we propose suggests that group 3 patients may experience auditory deterioration over time, eventually requiring CI. This deterioration may be accompanied by the appearance of retinal phenotypes that are more severe than expected^43^. Furthermore, therapeutic strategies in development for *USH2A*-related disorders^44–46^ include gene replacement, gene editing, and antisense oligonucleotide-based treatment. Clinical trials for *USH2A*-related RP using gene therapy are currently underway^47^, and the efficacy of gene therapy on hearing loss has been demonstrated in preclinical studies^48^. Here, we used WGS to identify two deep intronic variants that introduce cryptic splicing. These variants may be good candidates for therapy using antisense oligonucleotides, which prevent aberrant splicing and potentially rescue *USH2A*-related phenotypes. Notably, the use of allelic hierarchy data to predict clinical phenotypes may indirectly identify optimal therapeutic windows for gene therapy and other targeted treatments. An understanding of the impact of specific *USH2A* allele combinations on clinical presentation can guide the development of personalized treatment strategies and improve outcomes for affected individuals.

This study had some limitations that should be addressed in future studies. Our sample size, comprising 16 participants, was relatively small, thereby limiting the generalizability of our findings, both within the context of the South Korean population and beyond, particularly concerning individuals with *USH2A* variants. Nonetheless, to derive and augment such understanding, a meticulous analysis of clinical data and genotypes is imperative, even amidst the challenges posed by these rare cases. Future studies with larger sample sizes and more diverse patient populations are needed to validate and expand upon our findings. Additionally, our study lacked longitudinal analyses, and we did not elucidate how the *USH2A*-based allelic hierarchy operates over time. Future studies that include longitudinal follow-up and analyses of disease progression are needed to better understand the natural course of clinical phenotypes associated with *USH2A*-related disorders, as well as the impacts of particular genetic variants on disease progression. To assess potential progression, it is crucial to account for the concurrent presence of presbycusis or the gradual decline in lip-reading skills associated with visual deterioration. In our cohort, four participants aged 40 or above were identified as susceptible to presbycusis. Unfortunately, speech assessments were not administered to these individuals, precluding an evaluation of lip-reading. This is another limitation of our study. Despite these limitations, this study provides important evidence for an *USH2A*-related allelic hierarchy in South Korean patients. Our observations will lead to a better understanding of the natural course of clinical phenotypes and will improve the use of genotype-based therapy in the current era of precision medicine.

## Methods

### Study participants

The study protocol was approved by the Institutional Review Board of Seoul National University Hospital (IRB-H-0905-041-281, and IRB-H-2202-045-1298). Written informed consent was obtained from the participants or their legal guardians, in accordance with the tenets of the Declaration of Helsinki. A retrospective review was performed using the in-house database for genetic hearing loss at Seoul National University Hospital. Genomic DNA from 419 probands was subjected to clinical exome sequencing or WGS, regardless of audiological phenotypes or inheritance patterns. Probands with *USH2A* biallelic variants, either compound heterozygotes or homozygotes, were included. In total, 16 participants (9 male individuals and 7 female individuals) from 14 unrelated South Korean families with *USH2A* biallelic variants (3.3%) were identified. The mean age at ascertainment was 25.5 years (range, 0.3–64 years). Demographics, clinical phenotypes, and genotypes were evaluated.

### Audiological phenotyping

We analyzed audiological phenotypes in terms of the following four parameters: degree of severity, onset of hearing impairment, symmetry of hearing loss, and progression observed during follow-up evaluations. Pure-tone audiometry was used to assess the hearing thresholds of participants at six different octave frequencies (0.25, 0.5, 1, 2, 4, and 8 kHz), considering the participant’s age. For participants who were aged < 3 years, the auditory steady-state response was used to evaluate thresholds at four different octave frequencies (0.5, 1, 2, and 4 kHz). To determine the presence of conductive components, particularly in younger participants, we used tympanometry (with 226 Hz and 1000 Hz probe tones) and measured the bone conduction auditory brainstem response. Hearing threshold means were calculated for the four frequencies (0.5, 1, 2, and 4 kHz); hearing levels were categorized as mild (20–40 dB), moderate (41–55 dB), moderate to severe (56–70 dB), severe (71–90 dB), or profound (> 90 dB), based on the mean thresholds. Subjective onset of hearing loss information was obtained from each participant’s medical records or determined using the date of the first audiogram. Hearing asymmetry was defined as a difference of ≥ 15 dB between the mean hearing thresholds for both ears at 500, 1000, 2000, and 4000 Hz on pure-tone audiometry; alternatively, it was determined using the auditory steady-state response.

### Ophthalmological phenotyping

Clinical ophthalmic examinations included best-corrected visual acuity, ERG, GVF, OCT, fundus photography, and FAF. All examinations were preceded by pupil dilation. ERG was conducted in accordance with International Society for Clinical Electrophysiology of Vision standards using a commercial ERG system (Retiport32; Roland Consult, Brandenburg an der Havel, Germany)^49^. GVF evaluations were performed by professionals, in accordance with standard protocols^50,51^. OCT data were obtained using the Heidelberg Spectralis OCT instrument (Heidelberg Engineering, Hemel Hempstead, UK). Fundus photographs and FAF data were obtained using the Optos retinal imaging system (Optos, Marlborough, MA, USA).

### Whole-exome sequencing

Genomic DNA extracted from peripheral blood samples were collected from probands and subjected to exome sequencing using the Sure Select 50 Mb Hybridization and Capture kit (Agilent Technologies, Santa Clara, CA, USA). A library was prepared following the manufacturer’s instructions and was paired-end sequenced using a NovaSeq 6000 sequencing system (Illumina, San Diego, CA, USA) with an average coverage depth of 100×. The paired-end read length was 100 bp, and sequence reads were aligned with the University of California Santa Cruz hg19 reference genome browser (https://genome.ucsc.edu/). Subsequent to this, data were processed using the Genome Analysis Toolkit (GATK), adhering to the best-practice pipeline for the identification of Single Nucleotide Variants (SNVs) and insertions/deletions (indels)^52^. Variant annotation was executed using the ANNOVAR program, leveraging sources such as the RefSeq gene set and gnomAD^53,54^. Candidate variants were selectively identified, prioritizing rare non-silent variants, which encompassed nonsynonymous SNVs, coding indels, and splicing variants. For additional filtration of ethnic-specific variants, Korea Reference Genome Database (KRGDB) and Korean Variant Archive (KOVA) databases were used^55,56^. Additionally, the Clinvar and HGMD databases were screened to ascertain whether or not candidate variants have been previously detected in other families^57,58^. As previously described^12,59–64^, thorough bioinformatics analysis was conducted with strict filtering to identify candidate variants associated with SNHL and/or RP. For phasing analysis, segregation of these variants was investigated via Sanger sequencing of parental DNA samples.

### Whole-genome sequencing

DNA libraries for WGS were generated using the TruSeq DNA PCR-Free kit (Illumina) from 1 µg of genomic DNA. WGS was conducted on the NovaSeq platform 6000 (Illumina) to generate 151-bp paired-end reads with an average coverage depth of 30×. Sequenced reads were mapped to the human reference genome (GRCh38) using the BWA-MEME algorithm^65,66^, and duplicated reads were removed using SAMBLASTER^67^. Base substitutions and short indels were identified using HaplotypeCaller^52^ and trelka2^68^. Variants identified by either of these tools were included in further analyses. Genomic rearrangements were identified using Delly^69^; the breakpoints of genomic rearrangements of interest were visually inspected and confirmed using Integrative Genomics Viewer^70^. For phasing analysis, segregation of these variants was investigated via Sanger sequencing of parental DNA samples.

### Statistical analyses

All analyses were performed using R software (ver. 4.2.2; R Development Core Team, Vienna, Austria). The age at onset of subjective hearing loss, age at ascertainment, and degree of hearing loss were identified for the different genotype groups. Hearing thresholds were determined for the different frequencies, and mean values were calculated for each group. The Kruskal–Wallis rank sum test was used to evaluate differences in hearing threshold at each frequency among the three groups. For each frequency, each participant’s mean hearing threshold from both ears was analyzed. Correlations between audiological and ophthalmic phenotypes were evaluated using participants whose ophthalmic phenotypes had been investigated. Visual acuity, ERG, and GVF evaluations were used to investigate ophthalmic phenotypes. The pure-tone hearing threshold was used to investigate audiological phenotypes. Correlations between ophthalmic test values and hearing threshold values were evaluated using Spearman rank correlation analysis.

### Supplementary Information

Supplementary Figure S1.

Supplementary Table 1.

Supplementary Table 2.

## Data Availability

Sequence variations were submitted to Clinvar with the accession code (SCV003844066-SCV003844069) https://www.ncbi.nlm.nih.gov/clinvar/submitters/508922. The authors confirm that the data supporting the findings of this study are available within the article and its supplementary materials. The data (Individual-level whole exome/genome sequencing) that support the findings of this study are available from the corresponding author (S-Y.L, maru4843@hanamil.net) upon reasonable request. Some data may not be made available because of privacy or ethical restrictions.
