# Supplementary material for: Allelic hierarchy for USH2A influences auditory and visual phenotypes in South Korean patients
Source: Sci Rep. 2023 Nov 19;13:20239. doi: 10.1038/s41598-023-47166-w (PMC10658080; doi:10.1038/s41598-023-47166-w)
Supplement: Supplementary file 1 — Supplementary Figure S1. [file 41598_2023_47166_MOESM1_ESM.pdf]

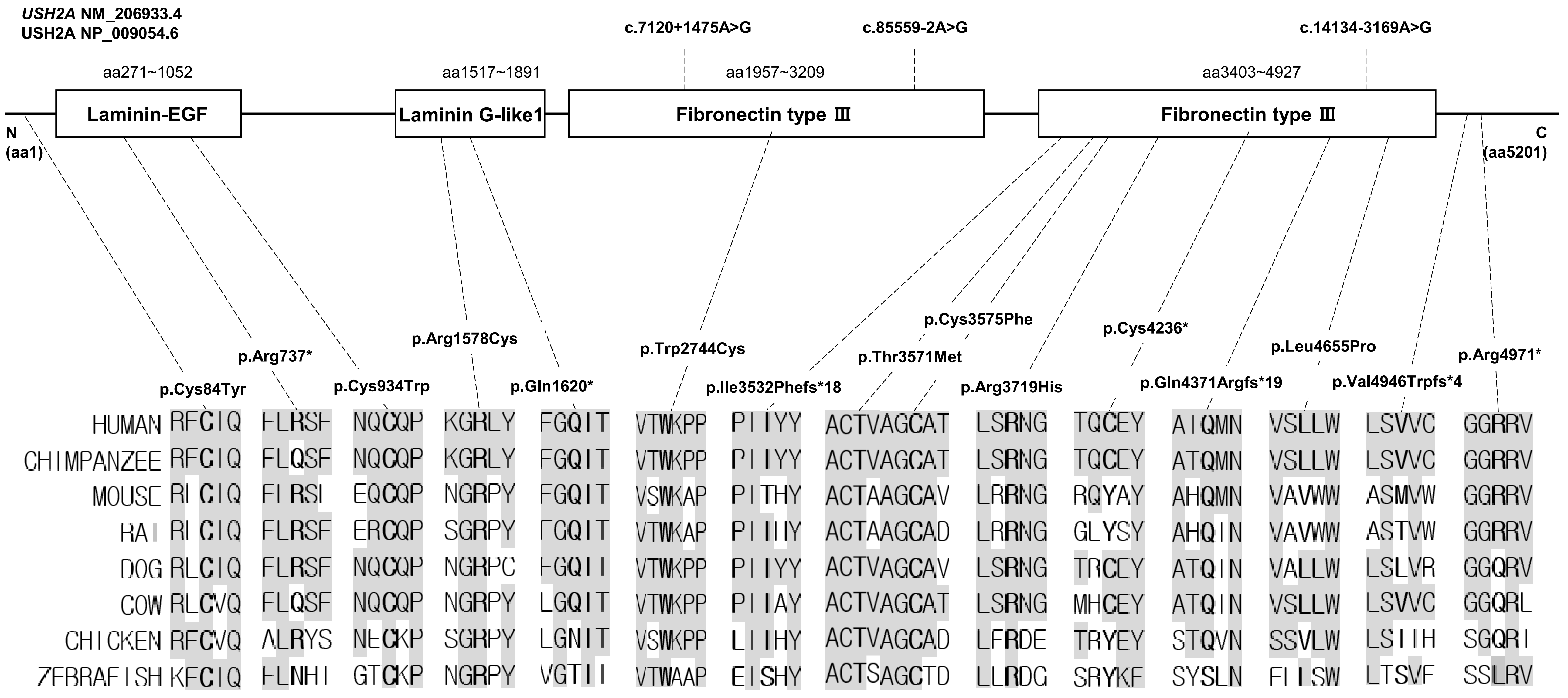

Supplementary Figure S1. Schematic representation of the USH2A protein domain structure. The affected residues in all USH2A variants identified in this study were conserved among different species. The sequence alignment of the usherin protein was constructed using the National Center for Biotechnology Information protein database (https://www.ncbi.nlm.nih.gov/protein), then visualized using the MAFFT online alignment program (https://mafft.cbrc.jp/alignment/server/index.html).
