# Supplementary material for: Allelic hierarchy for USH2A influences auditory and visual phenotypes in South Korean patients
Source: Sci Rep. 2023 Nov 19;13:20239. doi: 10.1038/s41598-023-47166-w (PMC10658080; doi:10.1038/s41598-023-47166-w)
Supplement: Supplementary file 2 — Supplementary Table 1. [file 41598_2023_47166_MOESM2_ESM.pdf]

***USH2A* (NM_206933.4)**

Genomic position	HGVS		Genomic position	HGVS	
(GRCh37/hg19)	nucleotide change	amino acid change	(GRCh38/hg38)	nucleotide change	amino acid change
Chr1:216595428C-T	c.251G>A	p.Cys84Tyr	Chr1:216422086C-T	c.251G>A	p.Cys84Tyr
Chr1:216420527G-A	c.2209C>T	p.Arg737*	Chr1:216247185G-A	c.2209C>T	p.Arg737*
Chr1:216419934A-C	c.2802T>G	p.Cys934Trp	Chr1:216246592A-C	c.2802T>G	p.Cys934Trp
Chr1:216270451G-A	c.4732C>T	p.Arg1578Cys	Chr1:216097109G-A	c.4732C>T	p.Arg1578Cys
Chr1:216262383G-A	c.4858C>T	p.Gln1620*	Chr1:216089040G-A	c.4858C>T	p.Gln1620*
Chr1:215963842T-C	c.7120+1475A>G	p.?	Chr1:215963842T-C	c.7120+1475A>G	p.?
Chr1:216052432C-G	c.8232G>C	p.Trp2744Cys	Chr1:215879090C-G	c.8232G>C	p.Trp2744Cys
Chr1:216051224T-C	c.8559-2A>G	p.?	Chr1:215877882T-C	c.8559-2A>G	p.?
Chr1:215955530A-T	c.10593del	P.Ile3532Phefs*18	Chr1:215782188TA-T	c.10593del	P.Ile3532Phefs*18
Chr1:215955412G-A	c.10712C>T	p.Thr3571Met	Chr1:215782070G-A	c.10712C>T	p.Thr3571Met
Chr1:215955400C-A	c.10724G>T	p.Cys3575Phe	Chr1:215782058C-A	c.10724G>T	p.Cys3575Phe
Chr1:215933077C-T	c.11156G>A	p.Arg3719His	Chr1:215759735C-T	c.11156G>A	p.Arg3719His
Chr1:215848545A-T	c.12708T>A	p.Cys4236*	Chr1:215675203A-T	c.12708T>A	p.Cys4236*
Chr1:215848137CATTT-C	c.13112_13115del	p.Gln4371Argfs*19	Chr1:215674795CATTT-C	c.13112_13115del	p.Gln4371Argfs*19
Chr1:215844483A-G	c.13964T>C	p.Leu4655Pro	Chr1:215671141A-G	c.13964T>C	p.Leu4655Pro
Chr1:215827321T-C	c.14134-3169A>G	p.?	Chr1:215653970T-C	c.14134-3169A>G	p.?
Chr1:215814032CA-A	c.14835del	p.Val4946Trpfs*4	Chr1:215640690CA-C	c.14835del	p.Val4946Trpfs*4
Chr1:215813957G-A	c.14911C>T	p.Arg4971*	Chr1:215640615G-A	c.14911C>T	p.Arg4971*

Refseq transcript accession number NM_206933.4; Refseq protein accession number NP_996816.3

Supplementary Table 1. The genotype information of the disease-causing USH2A variants, mapped to both hg19 and hg38
