# Supplementary material for: Allelic hierarchy for USH2A influences auditory and visual phenotypes in South Korean patients
Source: Sci Rep. 2023 Nov 19;13:20239. doi: 10.1038/s41598-023-47166-w (PMC10658080; doi:10.1038/s41598-023-47166-w)
Supplement: Supplementary file 3 — Supplementary Table 2. [file 41598_2023_47166_MOESM3_ESM.pdf]

Subject	Sex/<br>Age	Genotype	Variant type	Zygosity	Group	ACMG/AMP classification
SH351	F/6	c.14911C>T:p.Arg4971*;<br>c.2209C>T:p.Arg737*	Nonsense<br>Nonsense	Comp het	1	Pathogenic<br>Pathogenic
SH525	F/14	c.12708T>A:p.Cys4236*	Nonsense	Homo	1	Pathogenic
SH503	M/4M	c.14134-3169A>G;<br>c.14835del:p.Val4946Trpfs*4	Deep intronic (truncated)<br>Frameshift	Comp het	1	Pathogenic<br>Pathogenic
SH677-1	F/12	c.10593del:p.Ile3532Phefs*18;<br>c.8559-2A>G:p.?	Frameshift<br>Splicing (truncated)	Comp het	1	Pathogenic<br>Pathogenic
SH413	F/57	c.251G>A:p.Cys84Tyr;<br>c.13112_13115del:p.Gln4371Argfs*19	Missense<br>Frameshift	Comp het	2	Uncertain significance<br>Pathogenic
SH478	M/5	c.8559-2A>G:p.?;<br>c.11156G>A;p.Arg3719His	Splicing (truncated)<br>missense	Comp het	2	Pathogenic<br>Pathogenic
SH490	M/5M	c.2802T>G:p.Cys934Trp;<br>c.4858C>T:p.Gln1620*	Missense<br>Nonsense	Comp het	2	Likely pathogenic<br>Likely pathogenic
SH479	F/57	c.13964T>C:p.Leu4655Pro;<br>c.14835del:p.Val4946Trpfs*4	Missense<br>Frameshift	Comp het	2	Uncertain significance<br>Pathogenic
SH485-1	M/7	c.7120+1475A>G;<br>c.10712C>T:p.Thr3571Met	Deep intronic (truncated)<br>Missense	Comp het	2	Pathogenic<br>Pathogenic
SH485-2	F/5	c.7120+1475A>G;<br>c.10712C>T:p.Thr3571Met	Deep intronic (truncated)<br>missense	Comp het	2	Pathogenic<br>Pathogenic
SH608	M/47	c.2802T>G:p.Cys934Trp;<br>c.13112_13115del:p.Gln4371Argfs*19	Missense<br>Frameshift	Comp het	2	Likely pathogenic<br>Pathogenic
SH677-2	M/33	c.251G>A:p.Cys84Tyr;<br>c.8559-2A>G:p.?	Missense<br>Splicing (truncated)	Comp het	2	Uncertain significance<br>Pathogenic
SH325	M/27	c.11156G>A:p.Arg3719His;<br>c.8232G>C:p.Trp2744Cys	Missense<br>Missense	Comp het	3	Pathogenic<br>Pathogenic
SH767	M/34	c.2802T>G:p.Cys934Trp	Missense	Homo	3	Likely pathogenic
SH707	M/39	c.2802T>G:p.Cys934Trp;<br>c.4372C>T:p.Arg1578Cys	Missense<br>Missense	Comp het	3	Pathogenic<br>Pathogenic
SH637	F/64	c.10724G>T:p.Cys3575Phe;<br>c.11156G>A:p.Arg3719His	Missense<br>Missense	Comp het	3	Likely pathogenic<br>Pathogenic

Supplementary Table 2. Group classification of *USH2A* Genotypes and pathogenicity assessment according to ACMG/AMP Guideline
